# Supplementary material for: Rare Diseases in the Educational Field: Knowledge and Perceptions of Spanish Teachers
Source: Int J Environ Res Public Health. 2022 May 16;19(10):6057. doi: 10.3390/ijerph19106057 (PMC9140519; doi:10.3390/ijerph19106057)
Supplement: Supplementary file 1 [file ijerph-19-06057-s001.zip › ijerph-1670667-supplementary.pdf]

Prueba de KMO y Bartlett

Medida Kaiser-Meyer-Olkin de adecuación de muestreo		0.936
Prueba de esfericidad de Bartlett	Aprox. Chi-cuadrado	7699.083
	gl	190
	Sig.	0.000

Comunalidades

	Inicial	Extracción
IT1	1,000	0.666
IT2	1,000	0.664
IT3	1,000	0.631
IT4	1,000	0.664
IT5	1,000	0.492
IT6	1,000	0.674
IT7	1,000	0.755
IT8	1,000	0.609
IT9	1,000	0.785
IT10	1,000	0.728
IT11	1,000	0.699
IT12	1,000	0.695
IT13	1,000	0.728
IT14	1,000	0.782
IT15	1,000	0.748
IT16	1,000	0.740
IT17	1,000	0.570
IT18	1,000	0.668
IT19	1,000	0.771
IT20	1,000	0.633

Varianza total explicada

Componente	Autovalores iniciales			Sumas de cargas al cuadrado de la extracción			Sumas de cargas al cuadrado de la rotación		
	Total	% de varianza	% acumulado	Total	% de varianza	% acumulado	Total	% de varianza	% acumulado
1	9.474	47,372	47,372	9,474	47,372	47,372	4,229	21,147	21,147
2	2.253	11,264	58,636	2,253	11,264	58,636	3,720	18,598	39,745
3	1.084	5,421	64,057	1,084	5,421	64,057	3,673	18,365	58,110
4	0.887	4,436	68,493	0.887	4,436	68,493	2,077	10,383	68,493
5	0.756	3,779	72,272						
6	0.679	3,397	75,669						
7	0.611	3,055	78,724						
8	0.557	2,784	81,507						
9	0.523	2,616	84,124						
10	0.488	2,439	86,563						
11	0.394	1,972	88,535						
12	0.351	1,753	90,288						
13	0.329	1,643	91,931						
14	0.312	1,561	93,492						
15	0.264	1,320	94,812						
16	0.255	1,273	96,085						
17	0.226	1,131	97,216						
18	0.215	1,077	98,292						
19	0.191	0.954	99,246						
20	0.151	0.754	100,000						
